# Editorial: Exploring roles of diagnostic ultrasonography in veterinary medicine

**DOI:** 10.3389/fvets.2022.1084676

**Published:** 2022-11-18

**Authors:** Haney Samir, Ayman A. Swelum, Mohamed M. M. Kandiel

**Affiliations:** ^1^Department of Theriogenology, Faculty of Veterinary Medicine, Cairo University, Giza, Egypt; ^2^Department of Theriogenology, Faculty of Veterinary Medicine, Zagazig University, Zagazig, Egypt; ^3^Department of Theriogenology, Faculty of Veterinary Medicine, Benha University, Toukh, Egypt

**Keywords:** blood flow, diagnostic ultrasonography, doppler, fertility, cardiology, internal medicine, surgery, veterinary practices

Nowadays, ultrasound has become an important diagnostic tool, and its applications are proving more robust and tremendously beneficial in different aspects of veterinary practices. The advent of ultrasonography enables researchers and veterinarians to assess and monitor tremendous physiological and pathological events in the farm, companion, and wild animals (1). Because there is a growing demand for use of ultrasonography in studying, diagnosis, and monitoring many physiological events and diseases of animals, the goal of this special edition Research Topic is to shed light on the progress made in the past decade in applications of diagnostic ultrasonography in different aspects of physiological and pathological issues in the veterinary practices (reproduction, internal medicine, surgery, and cardiology). In this e-collection, 14 articles were published covering the aforementioned objectives and to provide direction and guidance to researchers in the field.

The study of Zhang et al. explored the usefulness of ultrasonography and Computer-assisted pixel assessment of the echotextural features of the mammary gland parenchyma in buffaloes during lactation at different somatic cell levels for diagnosing subclinical mastitis. In addition, this study found that examining the structures of the mammary gland parenchyma with vertical positioning showed better results than the horizontal direction. Similarly, in cows (2), it was reported that examining the vertical plane of the mammary gland was more suitable and could describe the significant correlation between echotextural variables and milk composition.

Tamura et al. described the usefulness of drip infusion cholangiography with computed tomography (DIC-CT) and abdominal ultrasonography for detecting the site of bile leakage in a dog with biloma [an encapsulated collection of bile outside or inside the biliary tract within the abdominal cavity; (3)]. Abdominal ultrasonography showed an enlargement of the gallbladder with the appearance of a kiwi-like pattern with residual central echogenic bile, indicating gallbladder mucocele. The gallbladder wall was not affected but the common biliary duct was dilated. An accumulation of well-defined anechoic fluid was observed around the right liver lobes. The amount of accumulated fluid was much larger compared to the ascites.

El-Sherbiny et al. reported the pivotal roles of pulsed Doppler ultrasonography for the assessment of testicular blood flow in rams in subtropical conditions. This study showed that treatment of heat-stressed rams with L-carnitine (LC) exerted a significant improvement in testicular blood perfusion because of reductions of Doppler indices (resistive index: RI and pulsatility index: PI), which explain the elevation of arterial blood perfusion and decrease of the vessel resistance to blood flow (4–6). In addition, it highlighted the pivotal roles of assessing the echotexture changes of testicular parenchyma in the control and LC-treated rams by computer image analysis (7, 8).

Daghash et al. studied the usefulness of B-mode and color Doppler ultrasonography to monitor the developmental growth and hemodynamic changes of corpus luteum (CL) in buffalos from Day 5 till Day 40 after ovulation in pregnant and non-pregnant buffaloes. This study showed a significant elevation in CL area from Day 20 to Day 40 in pregnant buffalos compared to the non-pregnant ones with a marked elevation of plasma progesterone levels in the same group, which could indicate the importance of adequate CL area and diameter to establish the pregnancy. Concomitantly, CL blood flow increased, as evidenced by lowering Doppler indices values (8), in pregnant females compared to those in non-pregnant ones.

Gremillet et al. reported the imaging findings in seven dogs and two cats with a presumptive diagnosis of sclerosing encapsulating peritonitis (SEP). SEP is defined as a chronic inflammatory condition in which the small intestines are encased in a dense fibro collagenous membrane (9, 10). Radiographs imaging showed abnormal shape and distribution of small bowel loops. By ultrasonography, free fluid was visible in all animals, either distributed in the entire peritoneal cavity (7/9) or focally collected (2/9). Peritoneal effusion was echogenic (6/9) and loculated or partially loculated by thin echogenic septations (3/9), suggesting an inflammatory process. Bowel loops had a corrugated appearance in all animals suggesting the occurrence of adhesions and bowel inflammation.

Hsu et al. presented the invaluable uses of different imaging modalities such as radiography, ultrasonography, and computed tomography (CT) for differential diagnosis and monitoring the treatment of a rare case of colonic intramural hematoma in cats. Abdominal ultrasonography revealed the existence of a local heteroechogenic intramural mass located between the submucosal and muscular layers of the descending colon, causing severe compression and closure of the lumen of the colon. Cranial to the intramural colonic mass, colonic lymph nodes were enlarged. For efficient differential diagnosis (with abscess, hematoma, and colonic intramural neoplasia), ultrasonography maybe not be a suitable tool. Scanning by CT and cytological examinations was performed to provide more information and reach the final definitive diagnosis. The patient responded well to the surgical approach without recurrence.

Gomes et al. published an important case report of a golden retriever dog suffering from *Helicobacter spp*. gastritis with chronic vomiting. Abdominal ultrasonography revealed the presence of a cluster of multiple, round, and well-defined, hypoechoic foci of different sizes (~0.7–1.8 cm) surrounded by gas within the lumen of the stomach. The histopathology of fundic cup biopsies showed large lymphocytic aggregates with mild multifocal inflammation of lymphocytes and eosinophils with few *Helicobacter*-like organisms on the surface of the epithelium. After appropriate treatment, the vomiting and fundic lesions were resolved on ultrasonographic examination. This case represented novel gross morphologic findings for *Helicobacter* spp. gastritis in dogs that responded well to the appropriate therapy and also highlighted how early intervention with advanced imaging can aid in diagnosis and treatment.

In equine, Hepworth-Warren et al. reported moderate sensitivity (66.7%) and high specificity (92.3%) of thoracic ultrasonography (TUS) for the identification of bacterial pneumonia in adult horses utilizing a TUS score system. In this study, ultrasonography was scored utilizing a novel scoring system evaluating several comet tails lesions, the presence or absence of pleural effusion, and/or pulmonary consolidation in each intercostal space. Ultrasonographic scores were significantly (*P* = 0.01) higher in the diseased group (median = 126) than in the control group (median = 20). Hence, TUS appears to be an acceptable stand-alone imaging tool for the diagnosis of bacterial pneumonia in horses when radiography is not practical.

Two-dimensional shear wave elastography (2D SWE) is an ultrasound technique that uses the technique of acoustic radiation force impulse to provide a quantitative assessment of tissue stiffness. In recent years, increased interest in this technique has developed in veterinary medicine due to its non-invasive nature, wide availability, and low cost (11). Interestingly, the study of Toom et al. aimed to compare liver stiffness values between dogs with the closed extrahepatic portosystemic shunt (EHPSS) and those with multiple acquired portosystemic shunts (MAPSS) after gradual surgical attenuation and to evaluate whether SWV could be used to determine EHPSS closure. The mean 2D SWV between dogs with closed EHPSS and those with MAPSS did not differ significantly in this study. However, these results could serve as a baseline and reference for future studies.

In veterinary cardiology, 5 papers were published in this e-collection. Mandour et al. explored the feasibility and repeatability of the color M-mode echocardiography (CMME) approach to measure different variables of the intraventricular pressure difference (IVPD) and intraventricular pressure gradients (IVPG) in goats and to explore the effect of sedation on the measured variables. This is the first study of the quantitative measurement of the IVPD and IVPG in goats using a novel CMME technique. The findings of the study, in conjunction with conventional echocardiographic methods, might deepen understanding of the hemodynamic changes of the left ventricle in goats as well as other farm animals for further experimental and clinical studies.

Ma et al. aimed to assess the changes in cardiac function in experimental induced uremic cardiomyopathy (UC, induced diastolic dysfunction) rats and to assess the therapeutic efficacy of salvianolic acid B (Sal B) using IVPG and two-dimensional speckle tracking echocardiography (2DSTE) techniques. This study confirmed increased ventricular stiffness and fibrosis in UC rats which was potentially treated by Sal B through decreasing edema, inflammation, and fibrosis. However, Further studies may be needed to clarify the molecular mechanisms of Sal B for the treatment of UC.

Hirose et al. reported the usefulness of novel non-invasive assessment of the IVPD and IVPG by color M-mode echocardiography (CMME), a promising method in diagnosing diastolic function, before and after surgical occlusion of patent ductus arteriosus (PDA a congenital heart defect associated with increased preload) in dogs. The findings of this interesting study revealed matched response of IVPD and IVPG to the reduced preload rather than left ventricular relaxation. This result is an initial step in the clinical utility of CMME-derived IVPD and IVPG measurements in the diastolic function evaluation in dogs with PDA that warrants upcoming clinical investigations.

Yoshida et al. reported the pivotal roles of diagnostic imaging in the assessment and follow-up of the therapy of a case report of pulmonary thromboembolism (PTE) secondary to immune-mediated hemolytic anemia (IMHA) in cats. The echocardiographic assessment indicated an enlargement in the right ventricle and atrium, mild tricuspid regurgitation, and a recognized thrombus in the main pulmonary artery. Regenerative anemia and auto-agglutination tests were suggested on blood examination. Appropriate interventions using antithrombotic therapy and immunosuppressive therapy improved the case.

Enokizono et al. evaluated the cardiovascular effects of a single dose of intramuscular (IM) and intravenous (IV) administrations of pimobendane (Pim) in healthy dogs by monitoring the echocardiographic hemodynamic parameters for up to 120 min. This study indicated that IM injection of Pim enhanced left ventricular relaxation and contractility, causing vasodilation within a short time approximately in the same way as the IV route. However, further clinical studies should be performed to explore the effectiveness of IM injections of Pim in case of emergency.

## Author contributions

HS conceptualized the idea and wrote the manuscript. AS and MK reviewed the manuscript with the first author. All authors reviewed the manuscript.

## Conflict of interest

The authors declare that the research was conducted in the absence of any commercial or financial relationships that could be construed as a potential conflict of interest.

## Publisher's note

All claims expressed in this article are solely those of the authors and do not necessarily represent those of their affiliated organizations, or those of the publisher, the editors and the reviewers. Any product that may be evaluated in this article, or claim that may be made by its manufacturer, is not guaranteed or endorsed by the publisher.
